# Transmission Dynamics of Methicillin-Resistant *Staphylococcus aureus* in a Medical Intensive Care Unit in India

**DOI:** 10.1371/journal.pone.0020604

**Published:** 2011-07-05

**Authors:** Solomon Christopher, Rejina Mariam Verghis, Belavendra Antonisamy, Thuppal Varadachari Sowmyanarayanan, Kootallur Narayanan Brahmadathan, Gagandeep Kang, Ben Symons Cooper

**Affiliations:** 1 Department of Biostatistics, Christian Medical College, Vellore, India; 2 The N.I. Clinical Research Support Centre, The Royal Hospitals, Belfast, United Kingdom; 3 Mahidol-Oxford Tropical Medicine Research Unit, Faculty of Tropical Medicine, Mahidol University, Bangkok, Thailand; 4 Nuffield Department of Clinical Medicine, Centre for Clinical Vaccinology and Tropical Medicine, University of Oxford, Oxford, United Kingdom; 5 Department of Gastrointestinal Sciences, Christian Medical College, Vellore, India; 6 Department of Microbiology, Christian Medical College, Vellore, India; Dana-Farber Cancer Institute, United States of America

## Abstract

**Background:**

Methicillin-resistant *Staphylococcus aureus* (MRSA) is a global pathogen and an important but seldom investigated cause of morbidity and mortality in lower and middle-income countries where it can place a major burden on limited resources. Quantifying nosocomial transmission in resource-poor settings is difficult because molecular typing methods are prohibitively expensive. Mechanistic statistical models can overcome this problem with minimal cost. We analyse the transmission dynamics of MRSA in a hospital in south India using one such approach and provide conservative estimates of the organism's economic burden.

**Methods and Findings:**

Fifty months of MRSA infection data were collected retrospectively from a Medical Intensive Care Unit (MICU) in a tertiary hospital in Vellore, south India. Data were analysed using a previously described structured hidden Markov model.

Seventy-two patients developed MRSA infections and, of these, 49 (68%) died in the MICU. We estimated that 4.2% (95%CI 1.0, 19.0) of patients were MRSA-positive when admitted, that there were 0.39 MRSA infections per colonized patient month (0.06, 0.73), and that the ward-level reproduction number for MRSA was 0.42 (0.08, 2.04). Anti-MRSA antibiotic treatment costs alone averaged $124/patient, over three times the monthly income of more than 40% of the Indian population.

**Conclusions:**

Our analysis of routine data provides the first estimate of the nosocomial transmission potential of MRSA in India. The high levels of transmission estimated underline the need for cost-effective interventions to reduce MRSA transmission in hospital settings in low and middle income countries.

## Introduction

Methicillin-resistant *Staphylococcus aureus* (MRSA) is one of the most important nosocomial pathogens globally [1] and a major cause of morbidity and mortality in high risk wards such as intensive care units [2]. In some countries in Asia, MRSA accounts for more than 70% of nosocomial *S. aureus* isolates [3], [4]. However, there remains a paucity of information about MRSA from most of Asia [5]. In India, the few studies there have been suggest that the prevalence of MRSA in hospitals is rising, and nationally MRSA is now thought to account for about 30% of *S. aureus* infections in hospital [6], [7].

The spread of multi-drug resistant pathogens such as MRSA poses a particularly serious threat in resource-poor settings where associated morbidity and mortality may greatly exceed that seen in resource rich settings [8]. Moreover, since antibiotics of last resort such as vancomycin or linezolid may be prohibitively expensive in many such settings, infections caused by such organisms can be effectively untreatable [9]. However, epidemiological studies in such resource-poor settings are largely lacking [10], and there have been no documented attempts to quantify the nosocomial transmission of MRSA in India. Quantifying such transmission is important because in many parts of India there are high levels of community-associated MRSA and establishing the sources and sinks of MRSA infection is vital for setting infection control priorities. In healthcare settings with limited resources, however, extensive epidemiologic surveillance and molecular typing methods conventionally used to quantify the extent of hospital transmission are prohibitively expensive. Novel statistical methods can offer a highly economic alternative [11], [12]. Such approaches, which make use of mechanistic transmission models, have proved useful in quantifying the extent of patient-to-patient transmission and unravelling the transmission dynamics of such pathogens in developed countries [13]-[16] and have been shown to yield similar results to conventional molecular typing methods [12], [17], [18]. In this study, we describe the epidemiology of MRSA in a single high risk medical intensive care unit (MICU) and use one such mechanistic model to estimate key parameters for a model of MRSA transmission among patients admitted to the unit using routine data.

The statistical challenge in quantifying MRSA transmission from routine infection data arises from the fact that only a proportion of patients harbouring MRSA have symptomatic infections; most are colonized asymptomatically and the epidemic process can therefore only be partially observed. In the absence of extensive (and expensive) whole ward surveillance and molecular typing methods it is therefore difficult to know to what extent increases in MRSA prevalence are the result of hospital transmission as opposed to admissions of MRSA positive patients from the community. Previous work has shown how this problem can be overcome by making use of infection data to impute the unobserved colonization dynamics, and we present data and inferences based on these methods [12], [19].

## Methods

### Study setting

The study setting was an eleven bedded medical intensive care unit (MICU) within a 2,234 bedded tertiary care teaching hospital, Vellore, south India. The staff-to-patient ratio was either 1∶3 or 1∶2 depending on the shift and bed occupancy was 100% (or very close to 100%) throughout the study. The infection control policy remained unchanged over the study period and recommended hand hygiene before every patient contact using either alcohol hand rub (Sterillium®) or surgical spirit BP which was kept at each patient's bedside. Hand hygiene compliance was not audited. No screening to detect asymptomatic MRSA carriage was performed. A single bed physically separated within the MICU was allotted for isolation of known cases of MRSA infection. No other specific interventions for the control of MRSA were in place throughout the study period. For most of the study period, vancomycin was the first choice antibiotic for treating MRSA infections provided patients could afford it. Towards the end of the study period, teicoplanin was available in India and was preferred over vancomycin. This research was approved by the Institutional Review Board of the Christian Medical College, Vellore - 632 004, India.

### Data

Data consisted of monthly totals of new MRSA infections among patients admitted to the unit each month for a period of fifty months, from January 2003 to February 2007. For those patients identified with an MRSA infection , the date of admission, time from admission (in days) to onset of symptoms that led to screening for MRSA, days of hospitalization, status at discharge, admission diagnosis, and cost of antibiotics for treating MRSA infection were collected. Repeated infections on the same patient during the study period were not included. Infection was classified as nosocomial if the time from admission to onset of symptoms was greater than 48 hours. All data were collected retrospectively from the Hospital Infection Control Committee office as obtained from the medical microbiology alert notification and Medical Records department.

Patients with MRSA infections were retrospectively classified as predisposing or non-predisposing to MRSA infection based on admission diagnosis. Patients were considered to be predisposing if they had at least one of a pre-specified list of systemic diseases (such as diabetes, HIV infection, etc) or local conditions (such as acute renal failure, subarachnoid haemorrhage, etc). Otherwise they were considered non-disposing to MRSA infection [20].

### Laboratory methods

*S. aureus* was identified from clinical isolates using the slide coagulase test. If this was positive, presence of *S. aureus* was confirmed using the tube coagulase test and mannite. Methicillin resistance was detected using oxacillin disk diffusion, in accordance with the Clinical and Laboratory Standards Institute.

### Statistical analysis

Preliminary data analysis was performed to check for time trends, seasonality, discontinuities, outliers and autocorrelation using standard graphical methods.

A previously described structured hidden Markov model (HMM) was fitted to the MRSA infection data [19]. This model was used to account for the partially observed infection process, as only the fraction of colonized patients who were symptomatically infected is observed. The model allows the unobserved (or “hidden”) number of patients asymptomatically colonized with the organism at different time points to be estimated.

The term ‘structured’ signifies that the underlying Markov model is constructed based on a mechanistic transmission model where new infections are generated according to a mass action process at a rate *βC_t_S*_t_ */N*, where *β* is a transmission parameter to be estimated, N is the ward population size, *C_t_* the (unknown) number of patients colonized or infected with MRSA at time *t* (the hidden *state* of the Markov chain), and *S_t_* represents the remaining MRSA-free patients. This transmission model corresponds to the assumption that doubling the number of MRSA positive patients on the ward (which corresponds to the colonization pressure), would double the chance of a susceptible patient acquiring MRSA in a small time interval. The model also allows estimation of a probability, ν, that each patient is carrying the organism when admitted to the ward, and assumes a known discharge rate, µ, which has been directly estimated from data. The approach also calculates the probability of each possible value of *C_t_* at each time point.

The number of observed infections at time *t*, *Y_t_*, is assumed to depend (probabilistically) on the corresponding number colonized at that time, *C_t_*_,_ according to a Poisson distribution with mean* λ* *C_t:_*, where the infection rate, λ, is a parameter to be estimated. All analyses were performed using R, a free open source language and environment for statistical computing [21].

### Results

A total of 72 MRSA infections were observed during the study period, with an average of 1.44 cases per month. Of these, 56 (78%) were classified as nosocomial, 67% of infected patients were male, the mean (SD) age was 48.8 (18.4) years and 92% of these patients were mechanically ventilated. The median length of hospital stay (inter-quartile range) of these infected patients was 15.5 (10–27) days, compared to 6 (2.25 – 12) days for all patients combined. The average prevalence of MRSA infection in the MICU over the whole study period (i.e., the percentage of MICU patients known to have MRSA infections at some point during their stay) was 2.1%.

Among patients with symptomatic MRSA infection, mortality in the MICU was 68%. Mortality rates in the predisposing (systemic or local) and non-predisposing groups were 79.2% and 40.9% respectively (odds ratio 5.49, 95% CI: 1.61, 19.31).

### Transmission dynamics

**Figure 1:**
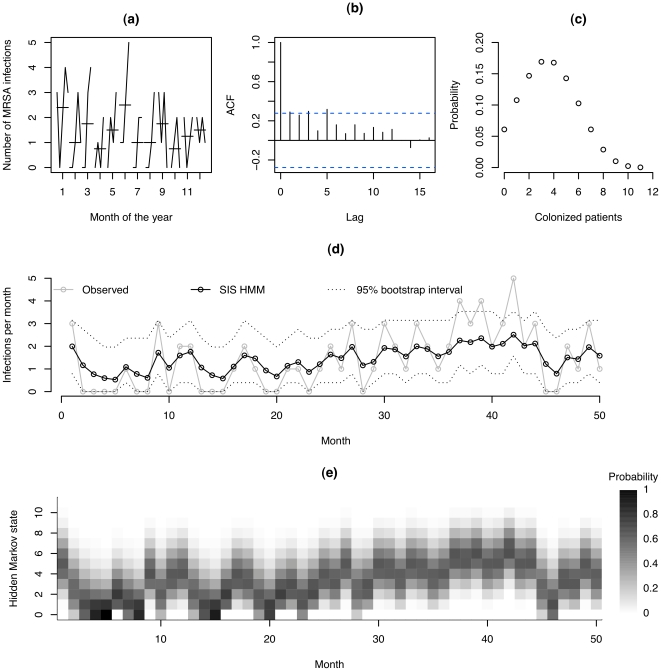
Preliminary analyses, and output from the structured hidden Markov model. (a) seasonal subseries plot showing the number of infections for each month of the year (horizontal lines represent the mean number of infections for the given month); (b) autocorrelogram, showing the correlation between recorded monthly infections in data points separated by 0,1,2,3 months (lags); (c) Stationary distribution of number of colonized patients; (d) Observed number of new infections and the expected number of new infections in each month estimated through the structured hidden Markov model using the entire series of observations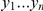. Broken lines indicate central 95% bootstrap intervals obtained by conditioning on the hidden state, and sampling this hidden state from a multinomial distribution using probabilities estimated by fitting the model to data (see Figure 1 (e)) with 1000 bootstrap replicates; (e) Estimated conditional probabilities of different hidden states over time, 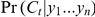.

The monthly count of MRSA infections in the MICU showed no apparent trend and there were neither obvious outliers nor discontinuities in the data. A seasonal subseries plot portraying the number of MRSA infections for the months of the year showed no pattern of high or low number of infections with reference to any particular month (figure 1(a)). Finally, the autocorrelogram, which shows the degree of correlation between infections at each time point and time points 0,1,2, … timesteps (lags) later, displayed a gradual decrease of auto-correlation with increase in lags: at the first three lags the autocorrelation was either significant at the 5% level or very close to being so, and no significant autocorrelation was detected at lags of six or more (figure 1(b)). Importantly, the autocorrelogram showed no pattern that was consistent with seasonal fluctuations. Thus, the time series was interpreted as being stationary.

The matrix of transitions in the number of new infections observed in consecutive months is displayed in table 1. The 0–0 transition occurred with the highest frequency, and the table shows that if there were no infections in one month, 50% of the time there were also no infections during the next month. Estimates of epidemiological parameters obtained with the transmission model are summarized in table 2. The estimated transmission rate parameter 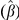 indicates that an MRSA positive patient in a fully occupied ward otherwise free of MRSA would have to stay on average 11.7 days (95% CI [2.4, 57.9]) before transmission of MRSA to another patient occurred (calculated as the reciprocal of the rate of new infections, *βCS_t_/N*). Assuming a mean length of stay for all MRSA patients (detected and undetected) of 5 days, the estimated ward-level reproduction number, *R_A_* (the mean number of secondary MRSA cases generated by one case during a single ward stay in an otherwise MRSA-free ward [19]) is 0.4 (95% CI [0.1,2.1]. The infection rate 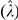 estimate indicates that the model predicts 0.4 (95% CI [0.1, 0.7]) symptomatic infections per colonized patient month (Table 2). The Akaike Information Criteria (AIC), which assesses goodness of model fit (the lower the better) for the HMM is 80.08 (as compared to the simple and widely-used Poisson model with AIC = 152.3). This indicates a greatly improved model fit using the dynamic HMM.

**Table 1:** Observed transition in the number of new infections in successive months.

Initial State	Number of transitions to the following stages					
	0	1	2	3	4	5
**0**	8	5	2	1	0	0
**1**	3	1	4	3	0	0
**2**	1	5	1	2	1	0
**3**	4	1	2	0	1	1
**4**	0	0	1	1	0	0
**5**	0	0	1	0	0	0

**Table 2:** Parameters estimated from the structured hidden Markov model.

Epidemiological Parameters	Model Estimates	95% Confidence Interval	
		Lower	Upper
Transmission rate, 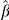 , (days ^−1^)	0.094	0.019	0.459
Positives on admission 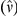	0.042	0.010	0.190
Infection rate 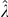 (months ^−1^)	0.393	0.056	0.731

Figure 1 (c) shows the stationary (unconditional) probability distribution of *C_t_*, which can be interpreted as the mean daily number of MRSA positive patients present on ward, and takes 12 possible values (the hidden states) from 0 to 11. This shows that the most likely number of MRSA positive patients on the ward at one time is 3–4, and 0 MRSA positive occurs with a probability of 0.061. Figure 1 (d) displays the observed number of new infections in grey (solid lines) overlaid with the expected number of new infections (given all observed data) fitted through the SIS HMM in black. Broken lines indicate 95% bootstrap intervals for this expectation obtained by conditioning on the hidden state, and sampling this hidden state from a multinomial distribution using probabilities estimated by fitting the model to data (these are shown in Figure 1 (e)). The figure shows a good fit between the observed and predicted number of infections providing graphical evidence for an appropriate transmission model.

### Costs

Besides the treatment cost for admission of the underlying disease condition, the additional cost due to treatment of MRSA infection was studied among patients with positive infection alone. The median total cost of MRSA treatment (cost of antibiotics alone) was 5,708.93 Indian Rupees (INR) (approximately $124), with an inter-quartile range of INR (2041.5, 22239.75) (approximately $45, $484). The median cost of treatment per day due to MRSA infection was 788.27 INR (approximately $17) resulting from additional antibiotic use to treat MRSA from the date of diagnosis of MRSA infection to the date of discharge or death.

## Discussion

Our analysis of a time series of MRSA infections provides evidence that substantial transmission of MRSA is occurring in the MICU, and yields estimates of basic transmission parameters. Such estimates are of value not just for helping to identify infection control priorities, but also for designing intervention studies and providing model-based assessments of the likely effectiveness and cost-effectiveness of different intervention policies. The results suggest that concerted infection control interventions would have the potential to substantially reduce the burden of MRSA disease in this setting. While resources were lacking to verify our findings using molecular typing methods, previous studies have found such transmission models and molecular approaches to yield very similar estimates [12]. We estimate a slightly higher incidence of disease per MRSA patient day in this setting that that estimated with the same approach from US data (0.39 versus 0.35 infections per colonized patient month) [19]. For comparison a recent study in 153 US veterans affairs hospitals [22], reported that pre-intervention infection rates in ICUs averaged 1.64 per 100 patient days. If we assume that 20% of the patient days were MRSA positive (which seems reasonable given that 13% were positive on admission) this corresponds to about 0.24 infections per month for an MRSA colonized patient. While this is 38% lower than the value we estimate in a resource-limited setting in India, we note that substantially elevated infection rates are consistently found in developing countries [23].

The stationary distribution reported in figure 1(d) shows the estimated probability of there being 0,1,2,… MRSA positive patients on the ward at one time. This shows that the estimated proportion of time with no colonization or infected patients is low (0.061). However, the data show that the proportion of months with no observed symptomatic infections is quite large (0.32). This discrepancy reflects a perpetuating hidden transmission and colonization that can result in infections as and when vulnerable patients come into contact with those who are colonized.

The costing data reported in this study suggest that the economic consequences of MRSA in this setting may be substantial. Based on 2005 figures, the median cost of anti-MRSA antibiotic treatment alone exceeded the quarterly earnings of more than 40% of the 1.2 billion people living in India [24]. The median daily cost of anti-MRSA antibiotics per day, 788.27 INR ($17) also represents a substantial proportion of the mean daily cost of MICU stay which was estimated from patient bills at 10,800 INR ($235). However, costing reported in this study must be regarded as both preliminary and almost certainly conservative: cost data amongst patients free of MRSA were not available for comparison and costs reported for MRSA patients include only the antibiotic cost and do not include costing for additional length stay resulting from MRSA infection. Some patients acquired more than one infection i.e., infection from other organisms in addition to MRSA and certain drugs that constituted for the antibiotic cost in this analysis were prescribed for treating infections from more than one organism and may not be specific to MRSA alone. On the other hand, a few patients with multiple infections including MRSA were not treated for MRSA and may have no additional cost of treatment due to MRSA infection. Importantly, since very few patients had medical insurance, ability to pay for expensive antibiotics was a crucial factor in deciding whether or not patients should receive a particular antibiotic or indeed any treatment at all. This resulted in a wide range of treatment costs for MRSA infections, and further highlights the importance of tackling antimicrobial resistance in parts of the world where effective antibiotics cannot be afforded by large parts of the population.

While this study does not rule out the possibility of substantial community transmission of MRSA in southern India, and although estimated transmission parameters are highly uncertain, the best parameter estimates demonstrate that substantial ongoing nosocomial transmission of MRSA is likely to be occurring in this region. As with any model, the validity of the findings will depend on the model assumptions which have been discussed at length elsewhere [19]. Two particular limitations of our study (and of the original hidden Markov model [19]) are the assumption of a constant rate of ward discharge (implying an exponential length of stay distribution) and the assumption that all autocorrelation is generated by the epidemic process. We lacked sufficient data to assess either assumption, though note that MICU length of stay distributions are typically right skewed with very long tails and a mode close to zero, and a constant rate of MICU discharge therefore seems a reasonable simplification. Moreover, while other factors affecting the transmission process cannot be ruled out, no specific interventions to reduce MRSA were implemented over the period when the data was collected. Thus, while we think these assumptions are plausible, and previous studies have shown good agreement with estimates of the same population parameters derived using molecular data [12], and model predictions show good agreement with our data, a major investment in microbiology and patient screening would be required to provide further validation for our findings.

Studies to investigate the control of MRSA and other healthcare associated infections in resource poor settings are now considered a priority by the World Health Organization [23]. In particular there is a need to investigate the value of enhanced surveillance for MRSA through patient screening, and to develop control policies that are cost-effective in low and middle income countries.
